# Supplementary material for: Comparison of the PF07598-Encoded Virulence-Modifying Proteins of L. interrogans and L. borgpetersenii
Source: Trop Med Infect Dis. 2022 Dec 26;8(1):14. doi: 10.3390/tropicalmed8010014 (PMC9863803; doi:10.3390/tropicalmed8010014)
Supplement: Supplementary file 1 [file tropicalmed-08-00014-s001.zip › tropicalmed-2068612-supplementary.pdf]

Supplementary file

Methods

Phylogenetic tree

To accomplish this tree, the evolutionary history was inferred using Neighbor-Joining method using MEGA 11 [45,46]. The bootstrap consensus tree inferred from 1000 replicates is taken to represent the evolutionary history of the taxa and the percentage of replicate tree in which the associated taxa clustered together in the bootstrap test (1000 replicates) are shown next to the branches the evolutionary distances were computed using the p-distance method and are in the units of the number of amino acid differences per site. Branches corresponding to partitions reproduced in less than 50% of bootstrap replicates are collapsed. There was a total of 270 positions in the final data set [46].

Amino acid composition

To identify the amino acid composition, the Expasy ProtParam (https://web.expasy.org/protparam/protparam-doc.html) [49] tool was used for VM protein, and the result was plotted, analyzed, and synchronized. To consolidate this information, the heatmap with amino acid content was constructed using the heatmap2 function of package gplots.

Data

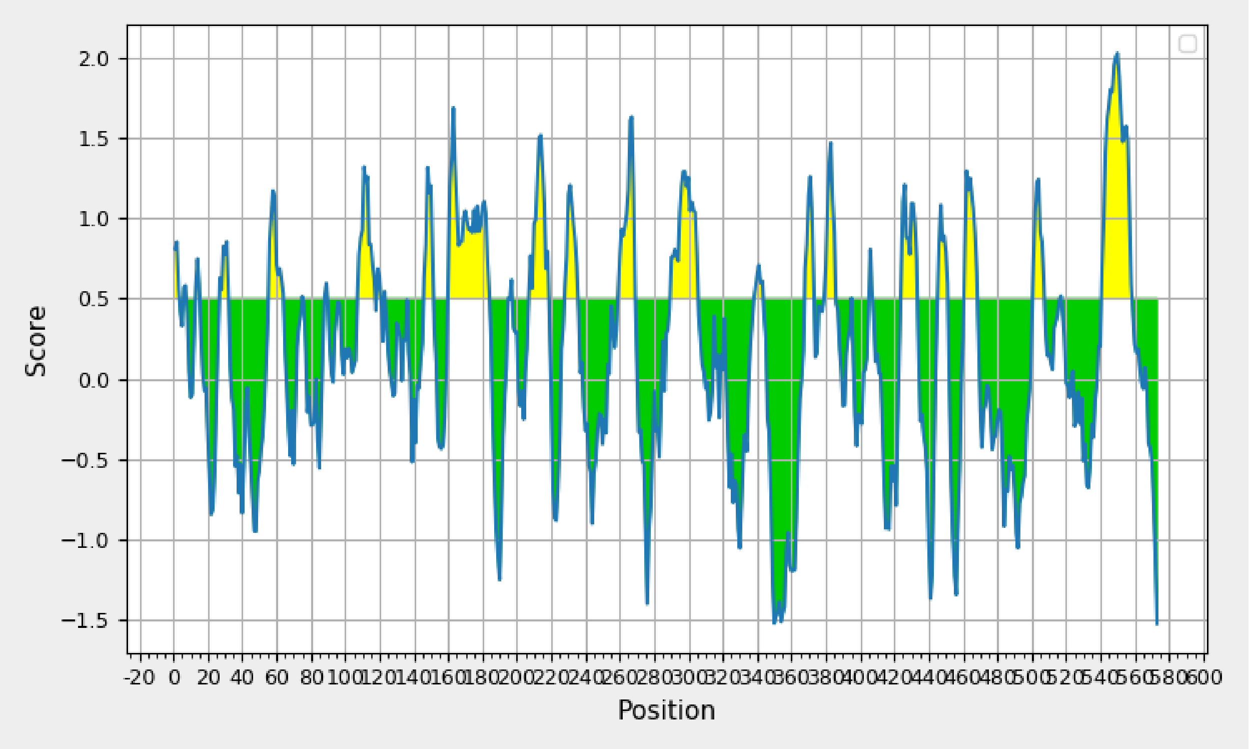

Supplementary Figure S1. Epitope analysis based on iedb.org from the amino acid sequence of *Leptospira interrogans* Lai, LA1400 which has the ortholog LIC_12340. The yellow color indicates possible epitope positions. Considerable analysis data: average (0.176), minimum (-0.001), and maximum (2.029).

Supplementary Table S1. Predicted peptides analysis of the amino acids sequence from *Leptospira interrogans* Lai, protein LA1400, based on iedb.org server.

No.	Start	End	Peptide	Length
1	1	3	MHG	3
2	6	7	NY	2
3	13	15	FTK	3
4	27	32	DNTAKA	6
5	56	64	TVVKGEETW	9
6	75	75	N	1
7	88	89	FW	2
8	108	117	RNSGDRYDHT	10
9	119	120	DS	2
10	123	123	D	1
11	146	151	QTTEGN	6
12	160	184	SSNKNTTPLYYNPESGHIAQYDPSS	25
13	195	195	T	1
14	197	197	K	1
15	207	218	KCSDAPIKKDNP	12
16	228	235	DKEGAITD	8
17	259	269	SYLEKDTTHSP	11
18	290	305	LGKTDQYCPAGNKESL	16
19	339	343	SGTIP	5
20	368	373	RGPLTS	6
21	379	385	DTAPNRD	7
22	395	395	P	1
23	406	407	NY	2
24	424	433	EWTSSDTITT	10
25	446	451	NSPPGS	6

No.	Start	End	Peptide	Length
26	461	468	NANGSTWG	8
27	501	507	PTMDPNE	7
28	517	517	T	1
29	541	558	RDCTGDGDGRRGSGRYPT	18

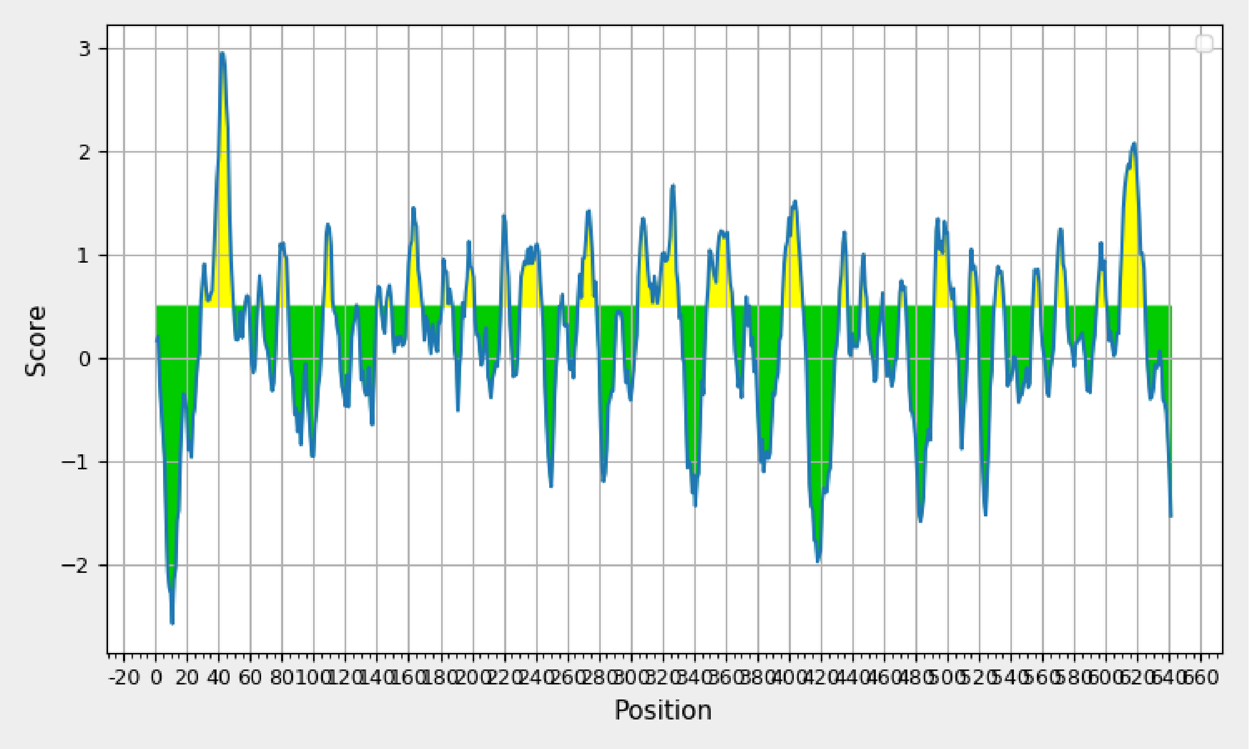

Supplementary Figure S2 Epitope analysis based on iedb.org from the amino acid sequence of *Leptospira interrogans* Lai, LA1402 which has the ortholog ortholog LIC_12339. The yellow color indicates possible epitopes positions. Considerable analysis data: average (0.211), minimum (0.003) and maximum (2.953).

Supplementary Table S2. Predicted peptides analysis of the amino acids sequence from *Leptospira interrogans* Lai, protein LA1402, based on iedb.org server.

No.	Start	End	Peptide	Length
1	29	49	SSKANYSIAQKPTDPPKDKPI	21
2	57	59	KTY	3
3	65	67	FSK	3
4	78	84	GDNTAKA	7
5	107	112	EPVVKG	6
6	127	127	N	1
7	140	142	FWT	3
8	146	149	KYRL	4
9	159	168	SKTSGDNYNH	10
10	182	187	ATPGNI	6
11	194	194	S	1
12	196	201	NSGWGD	6
13	219	223	GSSKE	5
14	231	244	NPESGHIAQYDPSS	14
15	255	255	T	1
16	257	257	K	1
17	267	278	KCSDAPIKKENP	12
18	305	330	KEGPNWGVAYTAKPSYLEKDTTHSPT	26
19	349	364	NLGKTDQYCPAGKKES	16
20	373	373	N	1
21	375	375	P	1
22	396	408	TFESANPGAIPQR	13
23	432	437	REPLTS	6
24	445	449	ASNRD	5
25	459	459	P	1

No.	Start	End	Peptide	Length
26	470	473	VDYA	4
27	492	504	EWESSSPLTTRSD	13
28	514	519	NSPPGS	6
29	530	535	ANGSIS	6
30	554	558	NMPTA	5
31	569	575	PTMDPNE	7
32	595	600	VGTYET	6
33	609	624	RDCTGDGDDRRGSGRY	16

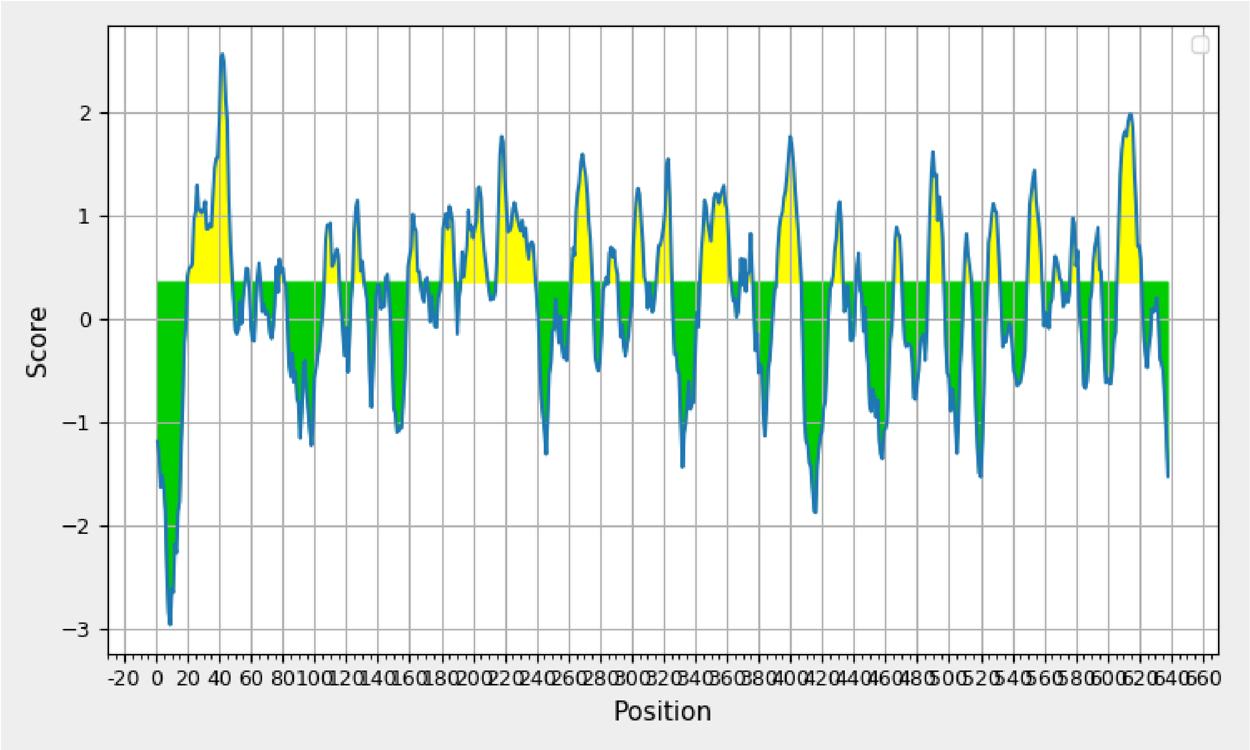

Supplementary Figure S3. Epitope analysis based on iedb.org from the amino acid sequence of *Leptospira borgpetersenii*, M6BGF1 which has the ortholog Q04V07. The yellow color indicates possible epitopes positions. Considerable analysis data: **average (**0.178), **minimum (**-0.009), **maximum (**2.560).

Supplementary Table S3. Predicted peptides analysis of the amino acids sequence from *Leptospira borgpetersenii*, protein M6BGF1, based on iedb.org server.

No.	Start	End	Peptide	Length
1	23	47	GSSISSSGIPGSVVQKPTDKPNDKP	25
2	65	65	G	1
3	76	76	C	1
4	78	78	D	1
5	107	115	KVIKGETNW	9
6	125	128	INNP	4
7	130	130	Q	1
8	159	164	KTSSDY	6
9	181	188	ATPGNVSV	8
10	193	193	A	1
11	195	208	DSNWGDGFWNGTPG	14
12	215	238	GGSNRNTTPIYYNPENGHLAQYNP	24
13	263	274	CSDAPVSSKENS	12
14	286	290	GGMIT	5
15	301	307	KTGPNWG	7
16	316	324	YLQADTTHS	9
17	344	361	GNLGNTEQYCPAGNKKNH	18
18	369	369	N	1
19	371	371	P	1
20	375	375	E	1
21	392	406	TYASARPGYRPQQAG	15
22	428	432	QRPLQ	5

No.	Start	End	Peptide	Length
23	442	443	ID	2
24	466	470	ATYAN	5
25	487	496	YEWESSPPIT	10
26	510	513	NSPT	4
27	525	531	RTDGTIS	7
28	550	558	NTPTTSFND	9
29	567	568	MN	2
30	577	581	EGPGR	5
31	591	594	PVGA	4
32	607	621	DCTGEGDDRRGSGRF	15

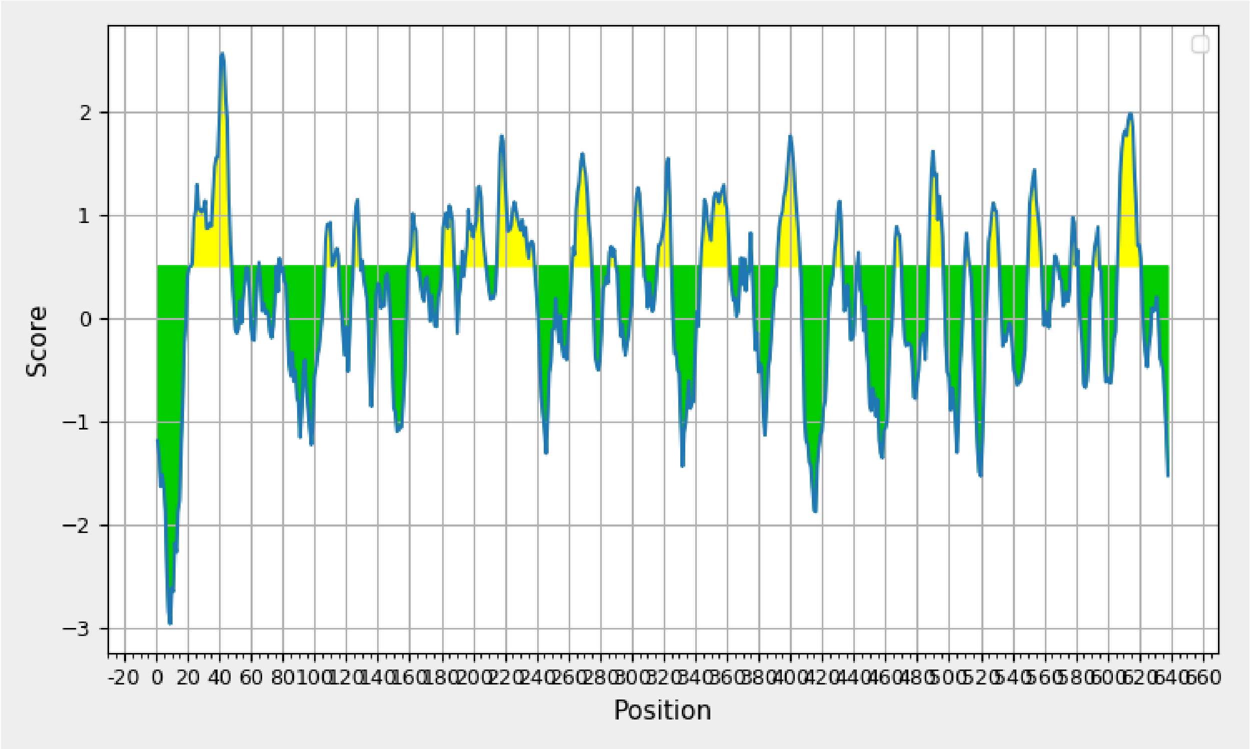

Supplementary Figure S4. Epitope analysis based on iedb.org from the amino acid sequence of *Leptospira borgpetersenii*, Q04V07 which has the ortholog M6BGF1. The yellow color indicates possible epitopes positions. Considerable analysis data: average (0.180), minimum (-0.009) and maximum (2.560).

Supplementary Table S4. Predicted peptides analysis of the amino acids sequence from *Leptospira borgpetersenii*, protein Q04V07, based on iedb.org server.

No.	Start	End	Peptide	Length
1	23	47	GSSISSSGIPGSVVQKPTDKPNDKP	25
2	65	65	G	1
3	76	76	C	1
4	78	78	D	1
5	107	115	KVIKGETNW	9
6	125	128	INNP	4
7	130	130	Q	1
8	159	164	KTSSDY	6
9	181	188	ATPGNVSV	8
10	193	193	A	1
11	195	208	DSNWGDGFWNGTPG	14
12	215	238	GGSNRNTTPIYYNPENGHLAQYNP	24
13	263	274	CSDAPVSSKENS	12
14	286	290	GGMIT	5
15	301	307	KTGPNWG	7
16	316	324	YLQADTTHS	9
17	344	361	GNLGNTEQYCPAGNKKNH	18
18	369	369	N	1
19	371	371	P	1
20	375	375	E	1
21	392	406	TYASARPGYRPQQAG	15
22	428	432	QRPLQ	5
23	442	443	ID	2
24	466	470	ATYAN	5
25	487	496	YEWESSPPIT	10
26	510	513	NSPT	4

No.	Start	End	Peptide	Length
27	525	531	RTDGTIS	7
28	550	558	NTPTTSFND	9
29	567	568	MN	2
30	577	581	EGPGR	5
31	591	594	PVGA	4
32	607	621	DCTGEGDDRRGSGRF	15

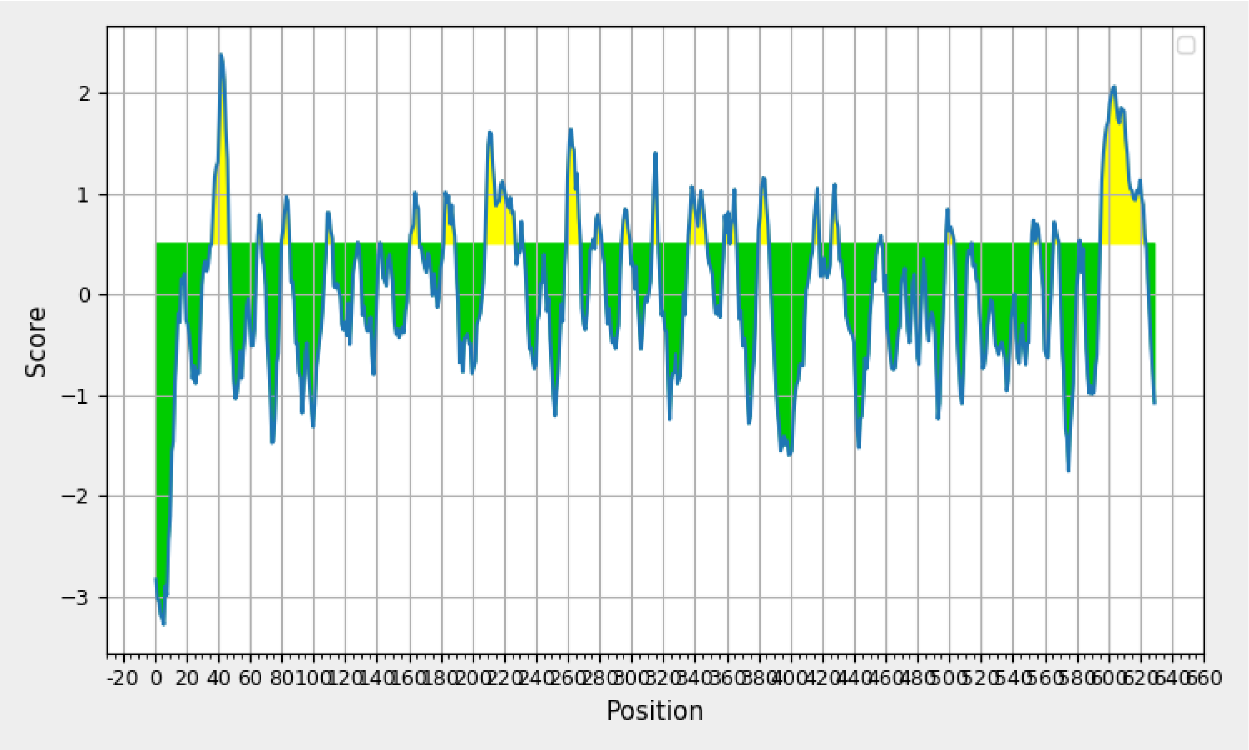

Supplementary Figure S5. Epitope analysis based on iedb.org from the amino acid sequence of *Leptospira borgpetersenii*, **Q04NE0** which has the ortholog **Q04T47**.
The yellow color indicates possible epitopes positions. Considerable analysis data: average (-0.013), minimum (-0.010) and maximum (2.375).

Supplementary Table S5. Predicted peptides analysis of the amino acids sequence from *Leptospira borgpetersenii*, protein Q04NE0, based on iedb.org server.

No.	Start	End	Peptide	Length
1	37	47	IQKPTDTPQDR	11
2	65	67	FSG	3
3	80	85	QDVSDA	6
4	109	112	SVVQ	4
5	128	128	N	1
6	142	142	W	1
7	161	166	KTSSDY	6
8	182	189	VSTPGNIS	8
9	209	227	GSKKNTTPIYYNPESGHLA	19
10	231	231	P	1
11	260	268	PISKDNPAY	9
12	276	276	E	1
13	278	281	GGML	4
14	294	299	YGPNWG	6
15	313	316	ATHS	4
16	336	347	GNLGKTEQYCPA	12
17	359	361	TLP	3
18	363	366	DFQL	4
19	380	386	SATPEAA	7
20	415	418	PLQS	4
21	426	430	ARDRD	5
22	457	457	G	1
23	498	503	HSPPGS	6
24	514	514	S	1

No.	Start	End	Peptide	Length
25	552	556	MPTTD	5
26	566	569	APDR	4
27	582	582	V	1
28	595	623	NCTGEGDDRRGTGEYPTSTSVNQCSGEGS	29

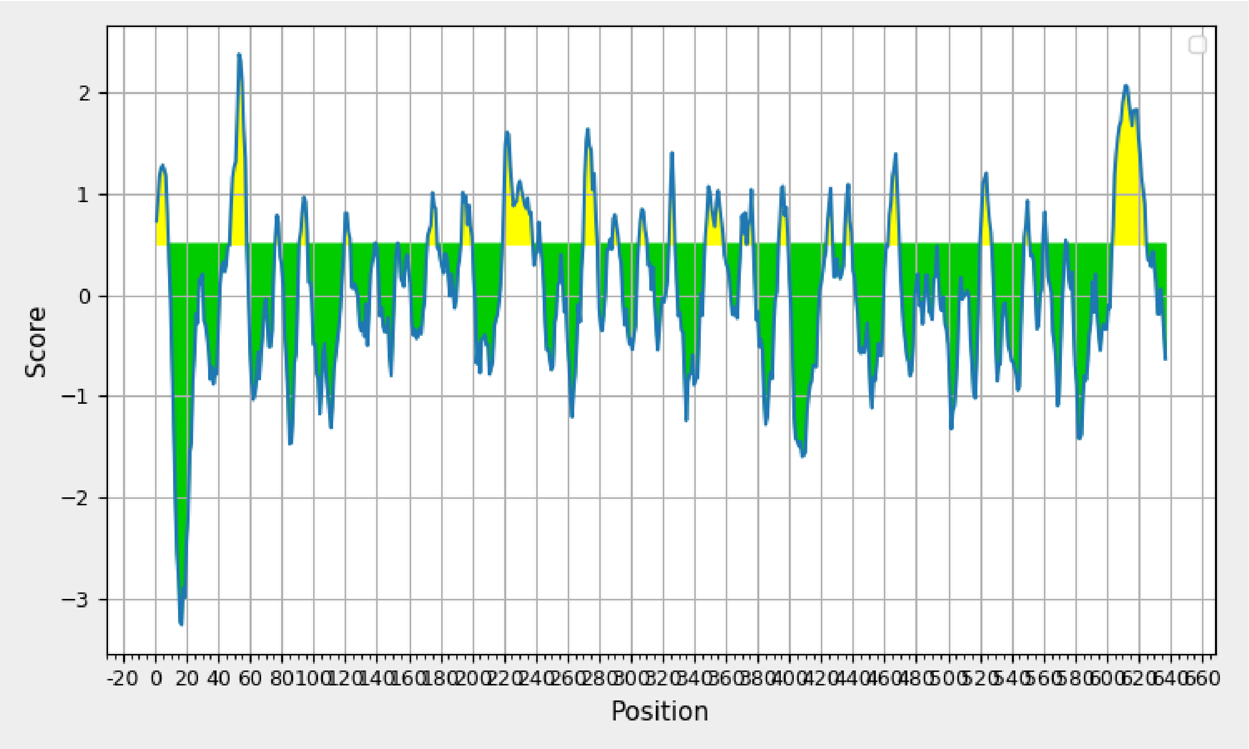

Supplementary Figure S6. Epitope analysis based on iedb.org from the amino acid sequence of *Leptospira borgpetersenii*, **Q04T47** which has the ortholog **Q04NE0**.The yellow color indicates possible epitopes positions. Considerable analysis data: average (0.025), minimum (-0.004) and maximum (2.375).

Supplementary Table S6. Predicted peptides analysis of the amino acids sequence from *Leptospira borgpetersenii*, protein Q04T47, based on iedb.org server.

No.	Start	End	Peptide	Length
1	1	8	MKNKKGEG	8
2	48	58	IQKPTDTPQDR	11
3	76	78	FSG	3
4	91	96	QDVSDA	6
5	120	123	SVVQ	4
6	139	139	N	1
7	153	153	W	1
8	172	177	KTSSDY	6
9	193	200	VSTPGNIS	8
10	220	238	GSKKNTTPIYYNPESGHLA	19
11	242	242	P	1
12	271	279	PISKDNPAY	9
13	287	287	E	1
14	289	292	GGML	4
15	305	310	YGPNWG	6
16	324	327	ATHS	4
17	347	358	GNLGKTEQYCPA	12
18	370	372	TLP	3
19	374	377	DFQL	4
20	394	399	DTAQGS	6
21	424	427	PLQS	4
22	435	439	ARDRD	5
23	463	469	TSAGDTN	7

No.	Start	End	Peptide	Length
24	520	527	RRRPDGTL	8
25	548	551	VPSA	4
26	560	562	LTP	3
27	574	574	E	1
28	604	624	NCTGEGDDRRGTGGYPAGTSV	21

Supplementary Table S7. Online tools used for analysis of *L. interrogans* Lai, Copenhageni and L. borgpetersenii.

Applications and tools	Website
Clustal Omega	www.ebi.ac.uk/Tools/msa/clustalo
Signal P 6.0	https://services.healthtech.dtu.dk/service.php?SignalP
Expasy ProtParam	https://web.expasy.org/protparam/protparam-doc.html
VaxiJen 2.0	http://www.ddg-pharmfac.net/vaxijen/VaxiJen/VaxiJen.html
Immune Epitope Database (IEDB)	http://www.iedb.org/
Jalview	https://www.jalview.org/
